# Applying Simulation Optimization to Minimize Drug Inventory Costs: A Study of a Case Outpatient Pharmacy

**DOI:** 10.3390/healthcare10030556

**Published:** 2022-03-16

**Authors:** Chia-Nan Chen, Chin-Hui Lai, Guan-Wei Lu, Ching-Chun Huang, Le-Jean Wu, Hui-Chuan Lin, Ping-Shun Chen

**Affiliations:** 1 Department of Pharmacy, Ditmanson Medical Foundation Chia-Yi Christian Hospital, East District, Chiayi City 600566, Taiwan; 03166@cych.org.tw (C.-N.C.); 03827@cych.org.tw (C.-C.H.); 01543@cych.org.tw (L.-J.W.); 02398@cych.org.tw (H.-C.L.); 2 Department of Information Management, Chung Yuan Christian University, Chung Li District, Taoyuan City 320314, Taiwan; chlai@cycu.edu.tw; 3 Department of Industrial and Systems Engineering, Chung Yuan Christian University, Chung Li District, Taoyuan City 320314, Taiwan; zerosevenwei@gmail.com

**Keywords:** inventory simulation, simulation optimization, inventory policy, two-stage clustering model, outpatient pharmacy

## Abstract

Drug inventory management is an important part of hospital management. The large amounts of drug data in hospitals bring challenges to optimizing the setting values for the safety stock and the maximum inventory of each drug. This study combined a two-stage clustering method with an inventory policy (*s*, *S*) and established a simulation optimization model for the case hospital’s outpatient pharmacy. This research used the simulation optimization software Arena OptQuest, developed by Rockwell Automation Inc (Rockwell Automation, Coraopolis, PA, USA), in order to determine the minimum and maximum values (*s*, *S*) of the best stock amounts for each drug under the considerations of cost and related inventory constraints. The research results showed that the minimum and maximum inventory settings for each drug in the simulation model were better than those set by the case outpatient pharmacy system. The average inventory cost was reduced by 55%, while the average inventory volume was reduced by 68%. The proposed method can improve management efficiency and inventory costs of hospital pharmacies without affecting patient services and increasing the inventory turnover rate of the drugs.

## 1. Introduction

Drug inventory management is an important part of hospital management [1,2,3,4,5]. Drug costs account for 20–25% of hospital management costs. Gebicki et al. [6] mentioned that drug costs account for a large part of hospital operating costs, second only to personnel wages. According to data from 2009 [6], the total drug expenditures of all hospitals in the United States was USD $27.7 billion, and the average expenditure per hospital exceeded USD $4.80 million, including the hospital’s medical quality and operating costs. Because Taiwan has a well-developed medical industry, well-established national health insurance, and advanced medical technologies, this study used the hospitals in Taiwan as a research target. According to previous years’ financial statements of medical consortiums, the Department of Medical Affairs, Ministry of Health and Welfare found that in the top 10 hospitals in Taiwan, the two most important medical costs in the past five years (2015–2019) were personnel expenses (about 37–49%) and drug expenses (13–25%), while the drug expenses accounted for about 20–25% of the total operating expenses. However, drug expenses included drug costs, drug inventory costs, and drug scrap costs. If the daily drug consumption can be effectively predicted, it is possible for a pharmacy drug manager to set a better drug purchase volume, maintain a reasonable drug inventory, and meet patients’ daily medication needs. If pharmacy drug managers can more accurately predict drug consumption, they can reduce drug purchases, drug inventory, and the quantity of drug scrapped, which can allow the hospital to effectively reduce drug expenses and increase hospital operating income. Therefore, hospitals have established relevant drug inventory performance indicators for the turnover rates of various drugs and drug inventory costs.

For drug management, a hospital’s pharmacy can be divided into a main pharmacy and subpharmacies. Usually, a hospital has only one main pharmacy (general pharmacy) with several subpharmacies, such as the emergency pharmacy, outpatient pharmacy, inpatient pharmacy, and chemotherapy pharmacy. Each patient’s drug use is recorded in the hospital information system (HIS) for each drug, including the patient’s medical record number, date of visit, gender, doctor code, drug code, drug name, usage, drug days, stock, medical department code, medical department name, medical department, whether usage is for a chronic disease prescription, whether usage needs to be reviewed in advance, the age of the patient, whether the drug is self-funded, and whether the drug is new. For example, if a patient receives three drugs, there would be three records in the HIS. Regarding daily drug inventory management, it is necessary to add the daily consumption statistics of a given drug (which is the total consumption of said drug by all patients per day) and determine the inventory level of the drug (that is, the maximum inventory and the safety stock) based on historical data on the daily consumption of the drug.

Improving inventory control is a critical issue in both hospitals and the manufacturing industry [7]. In order to improve management efficiency, many industries and scholars have used classification methods to classify drugs (or commodities) to distinguish the importance of the commodities and improve management intensity. Nigah et al. [8] mentioned that drugs can be classified into A, B, or C using the ABC classification method, based on the value or quantity of the drug, or into Vital, Essential, or Desirable using the VED classification method. Both methods classify drugs into different categories and show different degrees of importance among drugs, which then allows different management policies to be applied for drug inventory management.

In addition to distinguishing drugs for easy management, setting drug inventory quantities is a very important step. Good inventory settings can maintain stable inventory operations and ensure drug supply [9]. Common inventory strategies include a fixed order quantity system (*s*, *S*), a fixed cycle inventory system (*T*, *S*), the actual drug inventory order point (*s*), and order quantity (*S*), all of which are mostly set according to the experience or professional knowledge of the pharmacy administrator and the average patient demand. In terms of drug demand forecasting, linear time series forecasting methods have traditionally been used, including moving average (MA), exponential smoothing (ES), linear regression (LR), the autoregressive model (AR), and the autoregressive integrated moving average model (ARIMA). In addition, many studies have used heuristic algorithms or simulation methods to optimize drug inventory [4,10,11].

Some previous related studies have proposed optimized inventory models for individual drugs or specific types of drugs. Because the number of drugs analyzed in this study was large and these drugs had various types, it was difficult to develop inventory prediction models for each drug. It also takes more time to build models for a large number of drugs. This study established a simulation model of the outpatient pharmacy drug inventory system, which was aimed at the drug inventory operations of the case hospital’s outpatient pharmacy, to determine the best set values for optimal drug inventory strategy (*s*, *S*) that met the constraints. This study considered a large number of different types of drugs for inventory settings; thus, drugs with similar consumption patterns were first clustered as a group by using the *K*-means algorithm. For each drug cluster, this study identified the minimum value (*s*) and maximum value (*S*) of the best stock amounts for each drug, with the minimal total cost as the objective, by using the optimization simulation method. The simulations were performed using a scenario analysis, which considered the conditions of different scenarios to obtain the set values of the optimal maximum and minimum quantities of the stock of each drug in the corresponding scenario to help the pharmacy with drug inventory management and decision making. The proposed model can analyze a large number of different types of outpatient drugs and automatically determine the optimal drug inventory settings, thereby reducing the need for manual settings based on rules of thumb. According to this model, hospitals can also accurately control drug costs and effectively manage drug inventories.

In practical pharmacy drug management, as there are many drug items, how to formulate the maximum inventory and safety stock for each drug inventory strategy has always been an important issue [12,13]. Usually, the pharmacy drug manager or the subpharmacy team leader sets the maximum inventory quantity and safety stock quantity based on the average daily consumption of drugs and the drug delivery lead time of the pharmaceutical factory. For example, if the drug delivery lead time of a pharmaceutical factory is 2 days, then the drug safety stock quantity can be set to 3 days, and the maximum inventory quantity, to 4–6 days. The pharmacy drug manager or team leader finetunes the drug order quantity according to the current inventory of each inventory review or places a purchase order according to the predetermined drug order quantity. The pharmacy drug manager or team leader monitors the drug inventory status of each pharmacy drug according to the monthly pharmacy drug performance indicators, and if the performance target value is not reached, a strategy is adopted to reduce the ordering quantity of the drugs with more inventory. The adjustments in drug ordering are based on the rule of thumb of the managing director of pharmacy drugs or the team leader of each subpharmacy, and thus it is very difficult to effectively develop a hospital pharmacy drug management system. Further, pharmacy drug management can be conducted only based on experience, which is not conducive to the long-term development and management of the hospital organization.

In terms of the theoretical contributions, this study proposes a novel simulation model to analyze a large number of different types of drugs for inventory setting optimization. Because the *K*-means clustering algorithm was used in the model, it is possible to reduce the time needed to obtain the optimal solution of the inventory settings. Regarding the empirical contributions, the proposed model can effectively obtain the optimized values for the drug inventory setting. It also can help hospitals control the drug inventory and associated costs and enhance drug management efficiency.

The structure of this paper is as follows. Section 2 reviews the literature related to drug inventory management, including inventory cost, inventory strategy, and drug management, and the literature related to system simulation inventory optimization. Section 3 introduces the research methodology, clearly defines the research problem and the experimental process, explains the logical structure and parameter settings of this study, and presents the decision variables, objectives, constraints, and operational logistics of optimizing the drug inventory model. Section 4 shows the results of the discussion and analysis, provides information on the case hospital and its internal drug inventory operation situation, the completion of the validation and verification of the drug inventory system simulation model, and then the import of these data into the Arena OptQuest for model optimization (Rockwell Automation, Coraopolis, PA, USA). The corresponding objectives and constraints according to different scenarios are written. After the optimized solution is presented, the results of the current situation model are analyzed and compared with the results of the optimized solution model, and relevant discussions and suggestions are put forward. Section 5 offers the conclusion and suggestions for future research.

## 2. Literature Review

### 2.1. Inventory Control Management

Inventory plays an important role in the production and organization of logistical support in general enterprises. Inventories are managed in factories, wholesalers, retailers, and hospitals. In order to meet uncertain future demands, the business community stores appropriate quantities of materials, which is the purpose of the inventory. Fogarty and Hoffmann [14] pointed out that inventory management is a method developed in situations of uncertain demand and supply in time and quantity.

Inventory cost includes the following four items.

(1) Holding cost: The cost of storing items; the more stock there is, the higher the holding cost is.
(2) Storage cost: Inventory requires space, personnel, and equipment for storage, and these are storage costs.
(3) Ordering cost: The cost of placing an order for the company (or hospital). The total annual ordering cost is calculated by the processing cost per order multiplied by the total number of orders placed in the year.
(4) Out-of-stock cost: When the inventory cannot meet the demand and a loss is caused by a supply being out-of-stock, this loss is called the out-of-stock cost, which includes the cost of the emergency replenishment of the inventory, the loss due to the customer’s lost confidence in the company leading them to turn to others to buy substitutes, and the loss from a production line stopped because of a lack of materials. The costs incurred in this situation are intangible, meaning that they are difficult to directly calculate and are usually an approximated estimation.

By considering holding and ordering costs, Kelle et al. [15] established a drug inventory model to determine the minimum and maximum inventory (*s*, *S*) and then performed drug inventory control with the obtained results. The research results showed that this could reduce the costs of drug inventory by 70–80%.

Safety stock is additional inventory under anticipated demand, which is used to buffer random changes in the actual environment to prevent the occurrence of a shortage. Safety stock affects two types of costs: out-of-stock and holding costs. The higher the safety stock, the lower the chance of a drug shortage; conversely, the lower the safety stock, the greater the chance of a drug shortage. Because of the complexity of drug replenishment requirements and the difficulty of estimating the drug replenishment lead time of drug manufacturers, establishing a safety stock can reduce the risk of a drug shortage.

An inventory system is used for item inventory control and level maintenance. Its main purpose is to determine when replenishment is needed, what quantity needs to be replenished, and where to order goods from. Inventory review can be divided into two categories: (1) the perpetual ordering system, which is a fixed order quantity (*s*, *Q*) system, mostly adopts the quantitative ordering method for ordering; (2) the regular ordering system is to check the inventory at a fixed time and then decide on the order quantity; thus, it is also called the fixed order cycle (*T*, *S*) system.

### 2.2. Simulation Models for Inventory Control

The simulation models for pharmacy operations, drug inventory, and medical material inventory are organized as follows. Dong et al. [16] established an inventory simulation model with simulation software; verified the correctness of the model; compared two inventory strategies, (*R*, *S*) and (*Q*, *R*); and concluded that the (*R*, *S*) strategy was better than the (*Q*, *R*) strategy. Guerrero et al. [17] proposed a heuristic algorithm to solve the optimal order quantity of each product for the inventory strategy in a central drug warehouse, where model verification ensured that all strategy parameters were optimal. Their research compared the developed method with the inventory strategy of the case hospital and found that the inventory costs were reduced by about 45% while still maintaining a good level of service quality.

After studying the problem of drug shortages, Saedi et al. [10] constructed a mathematical planning model and enrolled a drug supplier and a hospital as the research subjects. The constraints for Saedi et al.’s study [10] included the delivery lead time (e.g., lead time = 0), meaning that it was not considered; the drug demand was random; and the demand arrival rate was subject to the Poisson distribution, meaning that the change time and length of change were random. There was supply disruption, and each drug was assumed to have a substitute. Thus, the continuous inventory review strategy was adopted, and the (*Q*, *R*) inventory strategy was used as the drug inventory strategy. That is, if the drug inventory was lower than the reorder quantity *R*, *Q* quantity would be ordered. This study proposed a two-stage heuristic algorithm to solve its mathematical programming model and calculate the cost, inventory space, drug shortage rate, and safety stock, and used the drug information of a hospital in Houston, Texas for verification.

As drug shortages affect the medication rights and health of patients, Azghandi et al. [11] studied the problem of drug shortages in the drug supply chain. The causes for a shortage of drug supply include external factors, such as an increase in demand for drugs, and internal factors, such as a drug recall by a pharmaceutical factory. Therefore, this study developed a mathematical simulation model to investigate the impact of drug recalls in different change scenarios (including the frequency and length of the drug supply fluctuation phenomenon) and used a data envelopment analysis (DEA) to determine the best inventory strategy. Nematollahi et al. [18] explored the multiobjective optimization problem of a drug supply chain by considering the profits of the supplier and distributor, as well as the patient service level (e.g., 1—drug shortage rate), where its multiobjective was to achieve the dual goals of maximizing profit under social considerations (e.g., the rate of drug availability), that is, the maximum profits of the supplier and the distributor. The research results showed that the centralized model considered that both the supplier and the distributor had better profits and patient service levels than the individual models of either the supplier or the distributor.

Buschiazzo et al. [1] studied the problem of optimizing the inventory of medical supplies for cardiac surgery. They considered procurement strategies (e.g., safety stock, available funds), actual warehouse space (e.g., warehouse capacity), and the characteristics of medical supplies (e.g., service life and service level) and suppliers (e.g., price, supply quantity, and minimum order quantity); used mathematical programming to construct a mixed integer programming model of cardiac surgery medical supplies inventory; and solved the problem of minimizing the total cost (including purchase cost and inventory cost) with AMPL and CPLEX. The results of mathematical planning were compared with the results of the system simulation, a related sensitivity analysis was performed, and the managerial implications of the research were discussed to increase the practical value of the results.

Chen et al. [19] studied the sales of low-price drugs (LPD) in the drug supply chain. The motivation for the research was that the government wanted to use legislation to ensure that patients could purchase LPD. Two strategies (the *P*-policy and *S*-policy) were proposed. The *P*-policy made decisions based on the ratio of purchase volume to expenses, while the *S*-policy made decisions based on the ratio of sales volume to profit. The study used inventory theory mathematics to derive the maximum profit from the entire drug supply chain and a guaranteed drug availability rate (that is, the probability that patients can buy LPD). Differentiation was used to find the extreme value and explored the maximum profit of the two policies. The results showed that the *P*-policy was better than the *S*-policy to create a win–win situation for the entire drug supply chain. Galli et al. [20] studied the drug inventory management of a ward through data exploration (e.g., *K*-nearest neighbors, decision tree, random forest, and XGBoot) and adopted the sample average approximation (SAA) method to evaluate the average absolute error value of drug demand under the random situation of drug demand to reduce the occurrence of emergency drug replenishment. The research results indicated that random forest and XGBoot were better than other methods. As simulation optimization has been applied in many studies with good results [1,3,21,22,23,24,25], this study used the simulation optimization method as the main research method.

### 2.3. Summary

Data relating to inventory costs, inventory strategy systems, and system simulation are discussed in the previous literature review. According to the clustering and classification literature, materials could be effectively distinguished through clustering or classification methods and using different management methods through different groups (or categories) could maximize the application of limited management resources. According to the inventory management literature, the inventory holding costs and out-of-stock costs have the greatest impact on inventory, and inventory strategies, safety stock, lead time, and other related knowledge are also relevant. In terms of inventory simulation, many documents have confirmed the effectiveness of inventory problems combined with demand forecasting and the clustering method in simulations. Simulation optimization can also be applied to solve various types of problems with good results. Therefore, this study combined the clustering method with an inventory model, and the simulation optimization method was used to solve the maximum and minimum drug inventory values under the considerations of cost and related inventory constraints.

## 3. Methodology

### 3.1. Problem Definition

Taiwan has a well-developed medical industry, well-established national health insurance, and advanced medical technologies. The medical systems in Taiwan’s hospitals have a complete collection of medically related data. This study observed the medical cost of Taiwan’s hospitals and found that drug purchase costs and management costs accounted for a relatively high proportion of overall costs [3,4,26]. Kelle et al. [15] and Gebicki et al. [6] also mentioned that drug costs accounted for a high percentage of a hospital’s operating costs; thus, if the drug inventory can be better managed, the hospital drug management costs can be effectively reduced. The hospital’s drug inventory setting value (*s*, *S*) is usually set as a fixed multiplier of the average drug consumption.

As mentioned in Section 2.1, inventory cost includes holding cost, storage cost, ordering cost, and out-of-stock cost. For pharmacy cost management, storage cost can be integrated into the holding cost. For patient safety issues, no drug shortage is allowed. The out-of-stock cost is then transformed from a cost item of the objective function to a constraint of the constructed simulation model. Therefore, only the holding cost and ordering cost were considered in this study. Usually, the holding cost and ordering cost can be calculated by using the activity-based costing (ABC) system or the time-driven activity-based costing (TDABC) system [27]. As the holding cost and ordering cost can be easily calculated based on the corresponding activities, this study used the ABC system to calculate these two costs in the objective function of the simulation model.

The demand for drugs occurs when doctors issue prescriptions for patients. As the number of patients changes daily, and drug prescriptions vary for each patient depending on their diagnosis, the consumption of each type of drug varies. Therefore, although setting the drug inventory at a fixed multiplier rate can achieve drug management, it may also lead to high inventory costs or drug shortage costs.

Per the literature, this study explored inventory (*s*, *S*) by considering the inventory costs. First, the drugs were divided into groups, the consumption of each drug was simulated, and the simulation model of each drug inventory was established. Next, the inventory costs were considered by simulation optimization to obtain the best inventory (*s*, *S*) for each drug.

### 3.2. Mathematical Model

This section introduces notations, an objective function, and corresponding constraints of the constructed mathematical model and the details. The details are as follows.

Notations:
Indices:
*p*: the index of drug no., where *p* = 1, 2, 3, …, *P*.
*t*: the index of time periods, where *t* = 0, 1, 2, 3, …, *T*.
Parameters:
$OC_{p}$: the ordering cost of drug no. *p*.
$HC_{p}$: the holding cost of drug no. *p*.
$DQ_{pt}$: the demand quantity of drug no. *p* at time period *t*.
$UP_{p}$: the upper bound of maximum quantity of drug no. *p*.
*NL*: the lower bound of number of orders for all drugs.
*NU*: the upper bound of number of orders for all drugs.
*M*: a huge positive constant.
Decision variables:
$X_{pt}$: the order quantity of drug no. *p* at time period *t*.
$Y_{pt}$: the indicator variable of order quantity of drug no. *p* at time period *t*;
$Y_{pt}$ = 1 if the pharmacy staff placed an order for drug no. *p* at time period *t* or 0 otherwise.
$I_{pt}$: the inventory of drug no. *i* at time period *t*.
$s_{p}$: the minimum quantity of drug no. *p*.
$S_{p}$: the maximum quantity of drug no. *p*.

Objective function: minimize cost

(1) $$\sum_{p}\sum_{t}HC_{p}\times I_{pt}+\sum_{p}\sum_{t}OC_{p}\times Y_{pt}$$

subject to:

(2) $$I_{p0}=0,\forall p=\{1,\;2,\;3,\;\ldots ,\;P\}$$

(3) $$I_{p(t-1)}+X_{pt}-DQ_{pt}=I_{pt},\forall p\in \{1,\;2,\;3,\;\ldots ,\;P\},\;\forall t\in \{1,\;2,\;3,\;\ldots ,\;T\}$$

(4) $$I_{pt}\;\geq \;0,\forall p\in \{1,\;2,\;3,\;\ldots ,\;P\},\;\forall t\in \{1,\;2,\;3,\;\ldots ,\;T\}$$

(5) $$X_{pt}\;\leq \;M\times Y_{pt},\forall p\in \{1,\;2,\;3,\;\ldots ,\;P\},\;\forall t\in \{1,\;2,\;3,\;\ldots ,\;T\}$$

(6) $$\sum_{p}\sum_{t}Y_{pt}\;\geq \;NL,\forall p\in \{1,\;2,\;3,\;\ldots ,\;P\},\;\forall t\in \{1,\;2,\;3,\;\ldots ,\;T\}$$

(7) $$\sum_{p}\sum_{t}Y_{pt}\;\leq \;NU,\forall p\in \{1,\;2,\;3,\;\ldots ,\;P\},\;\forall t\in \{1,\;2,\;3,\;\ldots ,\;T\}$$

(8) $$s_{p}\;\leq \;S_{p}-1,\forall p=\{1,\;2,\;3,\;\ldots ,\;P\}$$

(9) $$S_{p}\;\leq \;UP_{p},\forall p=\{1,\;2,\;3,\;\ldots ,\;P\}$$

(10) $$X_{pt}\;\in \;\mathrm{integer},\;\;\;\;\;\forall p\in \{1,\;2,\;3,\;\ldots ,\;P\},\;\forall t\in \{1,\;2,\;3,\;\ldots ,\;T\}$$

(11) $$I_{pt}\;\in \;\mathrm{integer},\;\;\;\;\;\forall p\in \{1,\;2,\;3,\;\ldots ,\;P\},\;\forall t\in \{1,\;2,\;3,\;\ldots ,\;T\}$$

(12) $$s_{p}\;\in \;\mathrm{integer},\;\;\;\;\;\forall p=\{1,\;2,\;3,\;\ldots ,\;P\}$$

(13) $$S_{p}\;\in \;\mathrm{integer},\;\;\;\;\;\forall p=\{1,\;2,\;3,\;\ldots ,\;P\}$$

(14) $$Y_{pt}\;\in \;\mathrm{binary},\;\;\;\;\;\forall p\in \{1,\;2,\;3,\;\ldots ,\;P\},\;\forall t\in \{1,\;2,\;3,\;\ldots ,\;T\}$$

Equation (1) sums the holding cost and ordering cost of each drug for each time period. Equation (2) represents the inventory of each drug being set to zero at time period 0. Equation (3) represents the flow conservation of each drug for all time periods. Equation (4) represents that no drug shortage is allowed for any time period. Equation (5) represents the relationship of the behavior in placing an order and its order quantity. Equations (6) and (7) represent the lower and upper bounds of the total number of orders placed for all drugs, respectively. Equation (8) represents that the minimum quantity of each drug is less than the maximum quantity of each drug. Equation (9) represents that the maximum quantity of each drug has its upper bound. Equations (10)–(13) represent the decision variables as integers. Equation (14) represents that the indicator variable is binary.

### 3.3. Research Process

**Figure 1:**
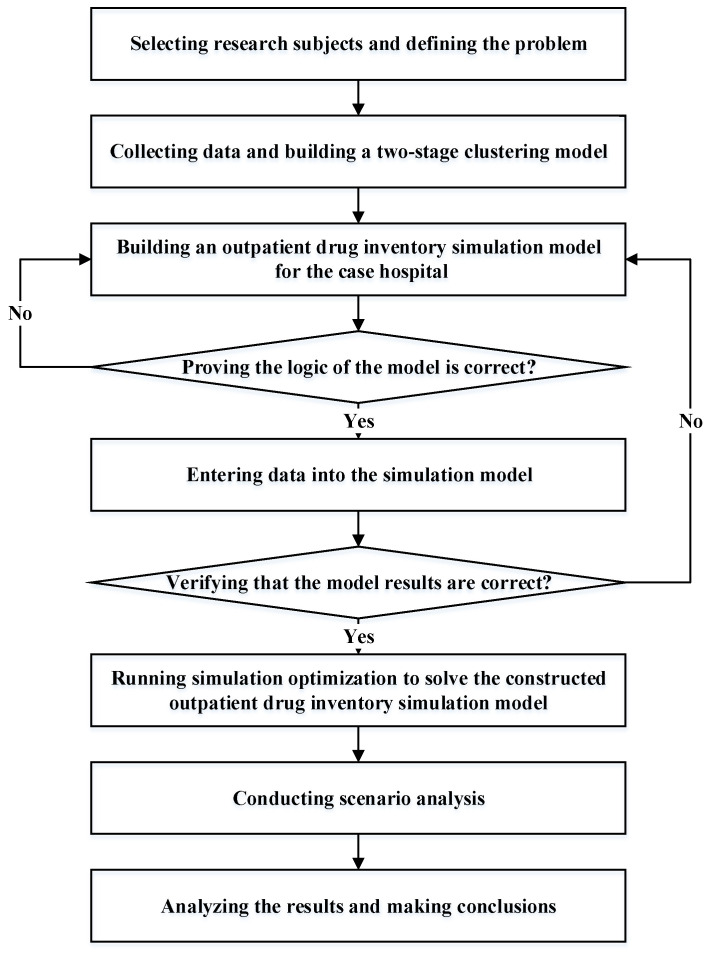
Research steps of this study.

Figure 1 shows the research steps of this research. First, the research subjects were selected, and the problem was defined. As the case hospital comprised a main pharmacy and multiple subpharmacies, this study focused on the outpatient pharmacy with the largest consumption ratio. Moreover, this study did not consider the replenishment behavior or emergency picking behavior among other subpharmacies and instead studied only the drug consumption and inventory (*s*, *S*) of the outpatient pharmacy. In practice, more than 1000 types of drugs exist for an outpatient pharmacy. This study focused mainly on the drugs paid for by the Taiwan Health Insurance System, which included about 700 drugs. This study collected one-year records on the drugs received by patients in the outpatient pharmacy. First, this study applied the Pareto principle (also called the 80/20 rule) to identify the key drugs with a high consumption quantity; those drugs were then divided into groups through a two-stage clustering method. This study used five indicators as input variables for the two-stage clustering method.

After the drugs were determined, this study began to establish an outpatient drug inventory simulation model. Using the Arena Input Analyzer software, the study performed data fitting on the daily consumption of drugs to determine the probability distribution of the daily consumption of each drug and established the outpatient drug inventory simulation model of the case hospital. The clustering method was adopted to classify drugs with similar consumption patterns into the same group. The best inventory value of the drugs in the same group was simulated to determine whether the drugs in the same group had similar inventory rules. Therefore, the clustering method is very important in determining the number of groups. This study used a two-stage clustering method to determine the setting of the number of groups, where the data for specific drugs included the (1) average monthly consumption, (2) monthly average number of prescriptions, (3) monthly drug consumption slope, (4) absolute value of the monthly average deviation rate, and (5) chronic disease prescription ratio. The details are introduced in Section 4.2.

After grouping, this study used the set value of drug inventory (*s*, *S*) as the decision variable and the minimization of average drug inventory cost (holding cost) and drug replenishment cost (ordering cost), as the objective. This study established an outpatient drug inventory model for drugs of the same group in outpatient clinics, based on drug inventory constraint conditions. After the model was established, verification and validation were carried out to ensure that it was consistent with the current situation of the case hospital. After verification, this study checked whether the logic of the simulation model was correct as planned. If correct, the collected data were used in the model; otherwise, the model was revised until the model logic was correct. In order to implement validation, this study checked for differences between the simulated average inventory of drugs and the average inventory of the case hospital. If the results showed no significant difference, the model could represent the inventory system of the case hospital and be used to analyze the various scenarios. If the results were different, the simulation model was modified until the results were similar to data from the case hospital.

After successful verification and validation, this study aimed to solve the problem of the outpatient drug inventory simulation model. In this step, this study used simulation optimization (i.e., Arena OptQuest software) to solve the problem and searched for the best value of each drug inventory strategy (*s*, *S*). The Arena OptQuest software uses the tabu and scatter searches to find the best value for each drug inventory strategy (*s*, *S*). However, as the simulation software program is a fixed program code, its algorithm cannot be modified. Therefore, how to refine the system simulation optimization algorithm is not included in the research scope of this study.

After determining the optimal setting value of each drug inventory, this study conducted scenario analyses to determine the optimal setting value of each drug inventory under different conditions and then analyzed the impact of different scenarios on the best setting values of each drug inventory. In the next section, the data results are summarized, analyzed, and discussed in regard to their managerial implications to provide a reference for pharmacy drug inventory to the case hospital.

## 4. Result Discussion and Analysis

### 4.1. Case Hospital

The drug inventory of the case hospital can be divided into two areas: the main pharmacy and the subpharmacy. For this study, the drug inventory was divided into three sections for introduction: stored drug items, the drug ordering and replenishment process, and the drug inventory policy. The first part was the drug items in storage. The main pharmacy is the largest drug storage area in the hospital and is responsible for managing all the drugs for use in the hospital. The subpharmacies reference the different departments in the hospital, including the outpatient, inpatient, emergency, and chemotherapy departments, each of which has its own exclusive subpharmacy where only the drugs for use by that department itself are stored. In the drug ordering and replenishment process section, the main pharmacy is responsible for managing all drugs and ordering drugs directly from suppliers. The subpharmacy ordering and replenishment systems do not directly order from the supplier but apply to the main pharmacy for replenishment, which is called the replenishment process. Regarding the drug inventory policy, the main pharmacy and the subpharmacies use the same set of policies (*s*, *S*). When a drug stock is lower than the minimum inventory setting (*s*), the drug must be ordered, and the order quantity is the maximum inventory setting, *S*, minus the current stock of the drug.

The main pharmacy and subpharmacies of the case hospital use the same drug inventory information system, and the inventory of each drug is set with reference to two parameters, namely, the inventory day and the drug inventory multiplier. The inventory day means the number of days of inventory kept in the pharmacy for a specific drug based on the average daily consumption. For example, the inventory day of TRACE in Table 1 is 5, meaning that five times the average daily consumption of TRACE should be kept in inventory. In the case of the hospital drug inventory information system, the inventory day is multiplied by the average daily drug consumption, and the result is taken as the minimum drug inventory (*s*), while the specific drug inventory is multiplied by the times the minimum drug inventory is taken, which is the maximum drug inventory (*S*). These two values are used as the system default values. Furthermore, the drug inventory information system can accept inventory staff manually set the minimum and maximum inventories of drugs, and the drug inventory information system gives priority to the manual settings rather than the system default values, as shown in Table 1. Taking the data for the drug TRACT as an example, the average number of inventory days was 5, the inventory multiplier was 3, and the average daily consumption was 2534 pills; thus, according to its inventory policies, the minimum inventory was 12,670 pills (average number of inventory days × average daily consumption), and the maximum inventory was 38,010 pills (inventory multiplier × minimum inventory). The minimum manual inventory of 4900 drugs and the maximum manual inventory of 33,460 drugs are manually set based on the experience of the inventory staff, and there are no specific rules. Therefore, the research purpose of this study was the optimization of the drug inventory setting values.

**Figure 2:**
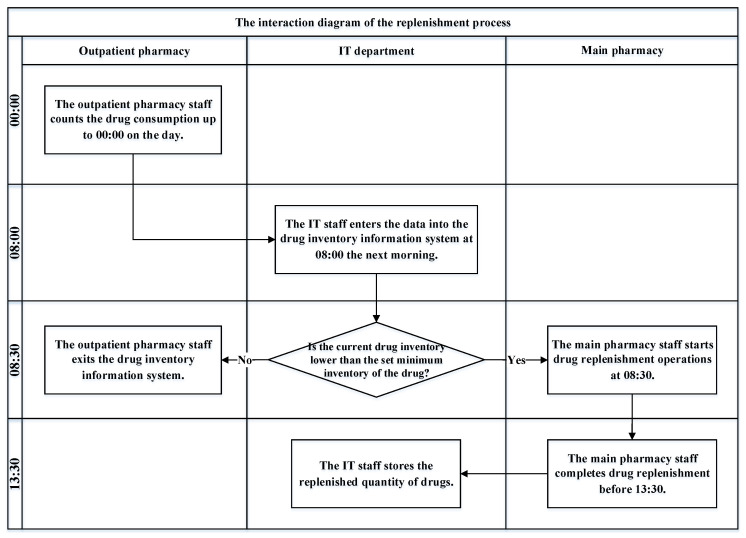
The replenishment process of the outpatient pharmacy of the case hospital.

In the replenishment process of the outpatient pharmacy of the case hospital, the outpatient pharmacy staff will count the drug consumption up to 00:00 on the day, and the information technology (IT) staff will enter the data into the drug inventory information system at 08:00 the next morning. The system will then determine whether the current drug inventory is lower than the set minimum inventory of the drug. If the inventory of a drug is lower than the set minimum inventory, the main pharmacy staff will start drug replenishment operations at 08:30 and complete the replenishment before 13:30. Then, the IT staff will input the replenished quantity of drugs into the information system for use by the outpatient pharmacy, as shown in Figure 2.

**Figure 3:**
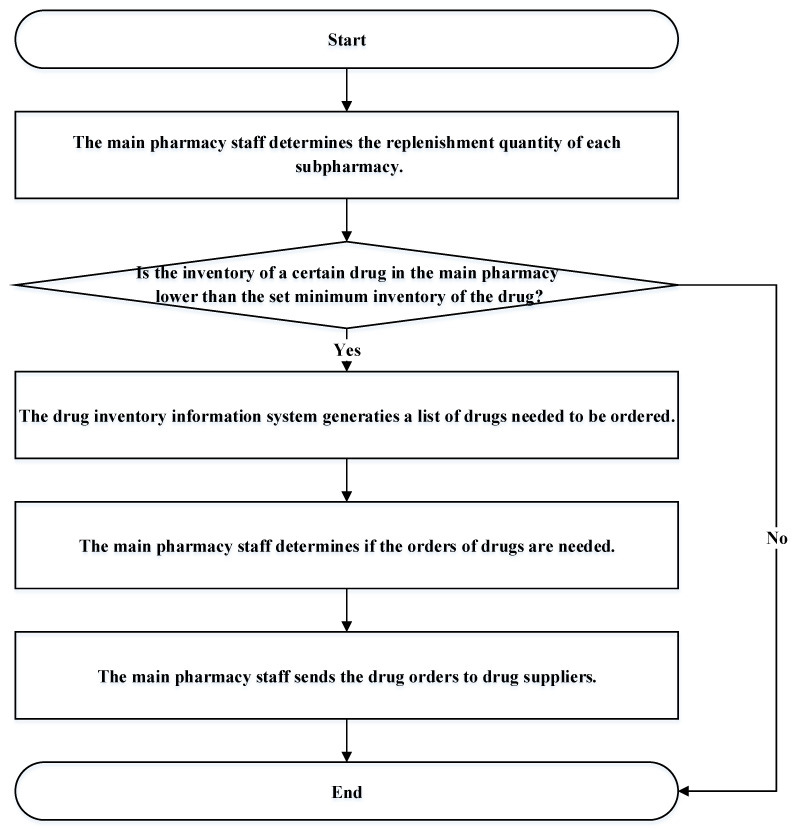
The main pharmacy ordering process.

Regarding the main pharmacy ordering process of the case hospital, first, the main pharmacy staff determine the replenishment quantity of each subpharmacy through the drug inventory information system to determine whether the inventory of a certain drug in the main pharmacy is lower than the set minimum inventory of the drug. If the drug inventory is higher than the set minimum inventory of the drug, an order is not made; conversely, if the drug’s inventory in the main pharmacy is lower than the set minimum drug inventory, the drug inventory information system generates a reminder that the drug needs to be ordered. Then, the main pharmacy staff determines the ordering quantity of the drug and places an order to the drug supplier, as shown in Figure 3.

**Figure 4:**
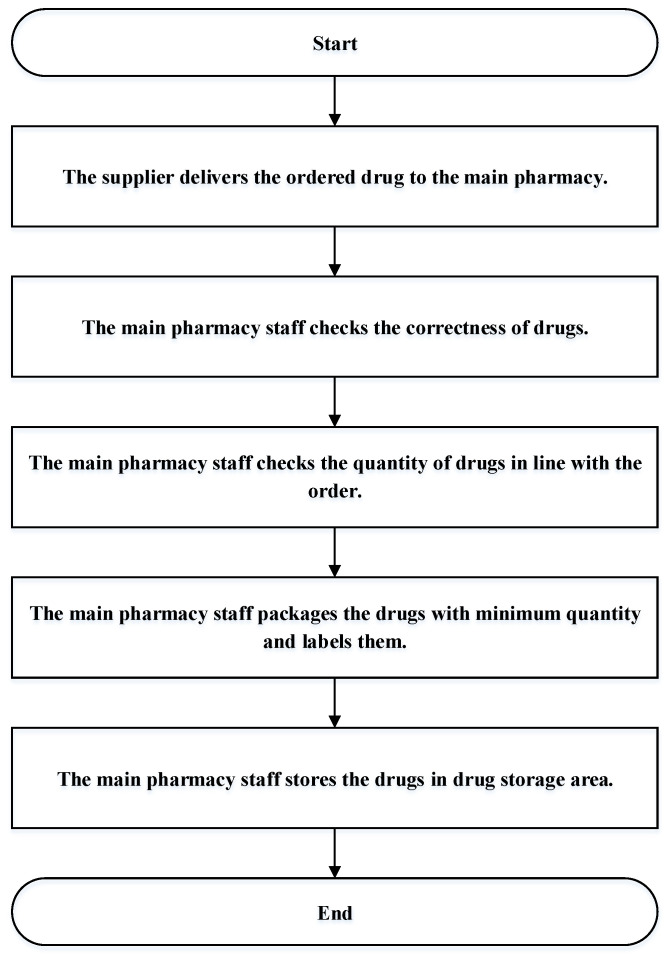
The replenishment process of the main pharmacy.

The replenishment process of the main pharmacy of the case hospital is that the supplier delivers the ordered drug to the inventory area of the main pharmacy within the specified time. Then, the main pharmacy staff checks the drug, including whether the correctness and quantity of the drug are in line with the order. The main pharmacy staff subpackage the drugs that pass the inspection into the minimum packaging quantity, as established in the hospital, and label them for ease of management. After these operations are completed, the drugs are stored in the main pharmacy, as shown in Figure 4.

### 4.2. Data Collection and the Two-Stage Clustering Method

This study collected data for all patients receiving drugs from the case outpatient pharmacy from January 2018 to December 2020. After analyzing the three years of data, the data between the first and second years were found not to be significant. However, the third-year data (2020) were impacted by the COVID-19 outbreak. Hence, this study used data from January to December 2019, which comprised about 3 million records, to build a simulation model for the case outpatient pharmacy. After excluding the items that did not need to be considered by the hospital and the drugs with missing data, there were 698 drugs used in the outpatient pharmacy. From the perspective of inventory management, the common rule for managing large inventory items is the Pareto principle (also called the 80/20 rule). This rule means that 20% of items account for about 80% of the total annual sales. The benefits of using the Pareto principle are that managers can monitor and control the inventory of a few critical items that account for the majority of total annual sales. In this study, the top 125 drugs accounted for 78.86% of the total annual sales, thereby qualifying for the Pareto principle. Therefore, this study selected the top 125 drugs used in the outpatient pharmacy as the study subject instead of studying all 698 drugs.

The monthly consumption data of these drugs were compiled, and five indicators were used as the input variables for the two-stage clustering method, which are detailed as follows.

(1) Average monthly consumption of a specific drug (AMC): Sum of the 12-month consumptions of one drug divided by 12 to obtain the average monthly consumption.
(2) Monthly average number of prescriptions of a specific drug (MANP): Sum of the 12-month number of prescriptions of one drug divided by 12 to obtain the monthly average number of prescriptions.
(3) Monthly drug consumption slope of a specific drug (MDCS): Sum of the difference between the monthly drug consumption and the average monthly drug consumption divided by the number of months.
(4) Absolute value of the monthly average deviation rate of a specific drug (AVMADR): The absolute value of the current month’s consumption of a specific drug minus the average monthly consumption of the drug, divided by the average monthly consumption of the drug.
(5) Chronic disease prescription ratio of a specific drug (CDPR): The amount of a specific drug prescribed for chronic disease patients divided by the amount of the drug prescribed for all patients.

**Figure 5:**
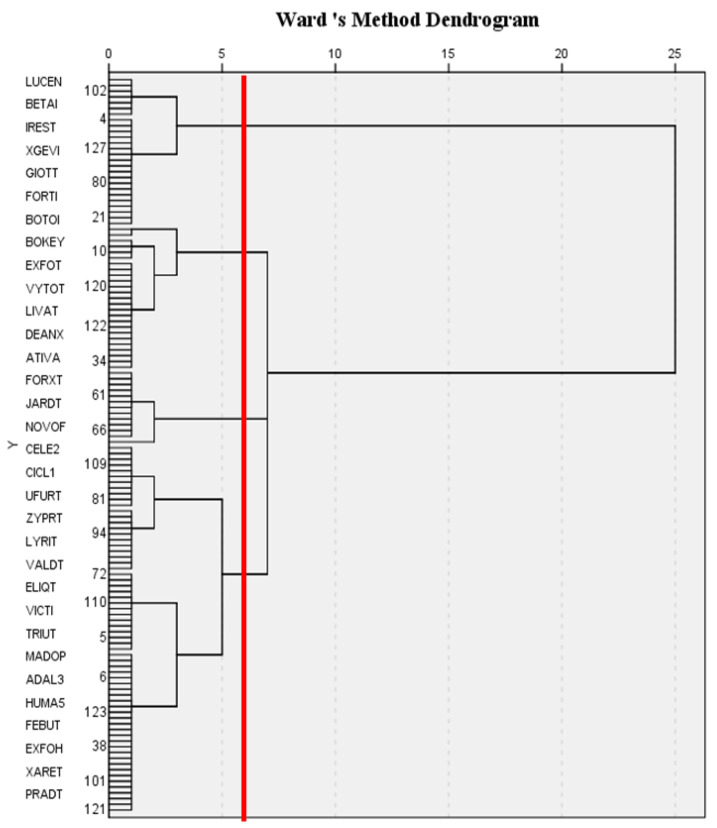
Using Ward’s method for hierarchical clustering.

This study used the SPSS statistics software, which includes a two-stage clustering method, in combination with the cohesive stratification method and the nonhierarchical clustering method for cluster analysis. In the first stage, this study used Ward’s method to calculate the distance and perform the cohesive stratification method, meaning that each datum was treated as a group, the distance between each group was calculated, and the two groups with the closest distance were combined into one group. The number of groups became less and less until all groups were combined into one group. This cohesive stratification method converted the results into a tree diagram (Figure 5) in which the *x*-axis represented the distance between two groups to form a new group and the *y*-axis represented the drug code. The cutoff point in the tree diagram had to be decided to determine the number of groups that are suitable for data grouping. There are two methods in the cutoff point selection principle: (1) determining the number of groups and then finding a suitable cutoff point and (2) finding places that are far apart as the cutoff points. The first method was adopted in this study to consider the number of groups, which were aimed to fall between 4 and 7. The *x*-axis in Figure 5 indicates that when the tangent point distance was set to 6, the tangent passed through four tree lines, meaning that the data could be divided into four groups; therefore, this study divided the data into four groups.

**Figure 6:**
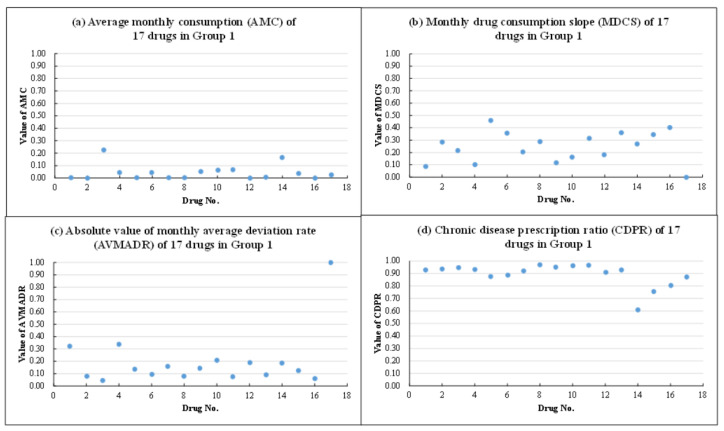
Four indicators of Group 1.

In the second stage, this study used the *K*-means method to divide the data into four groups. The *K*-means clustering method has been applied to various domains because it is simple and easy to use [28]. The four groups were named according to the four indicators of each group of drugs (i.e., average monthly consumption, monthly drug consumption slope, absolute value of the monthly average deviation rate, and chronic disease prescription ratio). Considering Group 1 as an example (Figure 6), when the average monthly consumption of drugs (Figure 6a) was low, this represented low consumption. The monthly drug consumption slope of drugs in Group 1 (Figure 6b) was mostly lower than 0.5, representing a recession. When the absolute value of the monthly average deviation rate of drugs (Figure 6c) was low, except for drug no. 17 (VIRET), this represented stability; when the chronic disease prescription ratio of drugs (Figure 6d) was high, this represented a high chronic disease prescription ratio. Therefore, the drugs in Group 1 belonged to the recession type, with stable, low consumption and a high chronic disease prescription ratio. The drug characteristics details of each group are introduced in the following.

(1) Drugs in Group 1 belonging to recession type, with stable, low consumption and high chronic disease prescription ratio: 17 drugs.
(2) Drugs in Group 2 belonging to growth type, with stable, low consumption and high chronic disease prescription ratio: 60 drugs.
(3) Drugs in Group 3 belonging to growth type with stable, high consumption and high chronic disease prescription ratio: 12 drugs.
(4) Drugs in Group 4 belonging to growth type, with divergent, low consumption and high nonchronic disease prescription ratio: 36 drugs.

### 4.3. Simulation Hypothesis

This study constructed the following four hypotheses for the outpatient drug inventory simulation model.

(1) It was supposed that the quantity of drugs in the main pharmacy could meet the replenishment needs of the subpharmacy for the outpatient clinic and that there would be no shortage of drugs. In practice, the quantity of drugs in the main pharmacy could meet the required replenishment quantity; therefore, this study assumed that the quantity for replenishment in the main pharmacy was sufficient.
(2) The inventory replenishment operations between the outpatient subpharmacy and other subpharmacies were not considered. In practice, when drug inventory management is performed by each subpharmacy, if the outpatient pharmacy does not have enough drugs during the nonreplenishment period but the drug inventories in other subpharmacies are sufficient, the outpatient pharmacy applies for drug replenishment from other subpharmacies to meet the current drug demand. As this study simulated only the drug inventory and consumption operations of the outpatient pharmacy with the highest consumption ratio and did not include other subpharmacies, this study did not consider the transfer operations among the subpharmacies.
(3) The emergent collection of drugs from the outpatient pharmacy was not considered. When the drugs in all subpharmacies are not enough to meet the needs of the outpatient pharmacy, even during the nonreplenishment period, the outpatient pharmacy still initiates an emergency request application to the main pharmacy, and the main pharmacy provides drugs to the outpatient pharmacy to meet its current demand for drugs. Since the emergent collection behavior does not often occur in the inventory management of the case hospital pharmacies, it is prone to occur only for certain drugs with a small inventory. Therefore, this study did not consider the rare occurrence of urgent drug collection operations.
(4) The effects of special holidays and long holidays were not considered in this study. As consecutive holidays and long holidays (such as annual holidays) in a year are special circumstances, it is necessary to estimate the consumption of drugs in advance for such days. As the frequency of special circumstances during the year is low, this model did not simulate special or long holidays and considered only fixed weekends, that is, two days off each week.

### 4.4. Simulation of Outpatient Drug Inventory Simulation Model

This study used a personal computer with Intel^®^ Core™ i7-8700 CPU, DDR4 64GB RAM, Windows 10 Professional version, and the simulation software Arena 16.0 and OptQuest toolbox, which were developed by Rockwell Automation Inc. (Rockwell Automation, Coraopolis, PA, USA). A system simulation model was established based on data for the current situation of the outpatient drug inventory in the case hospital. The simulation length of the model was set to one year, and the number of replications was set to 30. Furthermore, the number of simulation optimization iterations to be run by Arena OptQuest was set to 1200.

**Figure 7:**
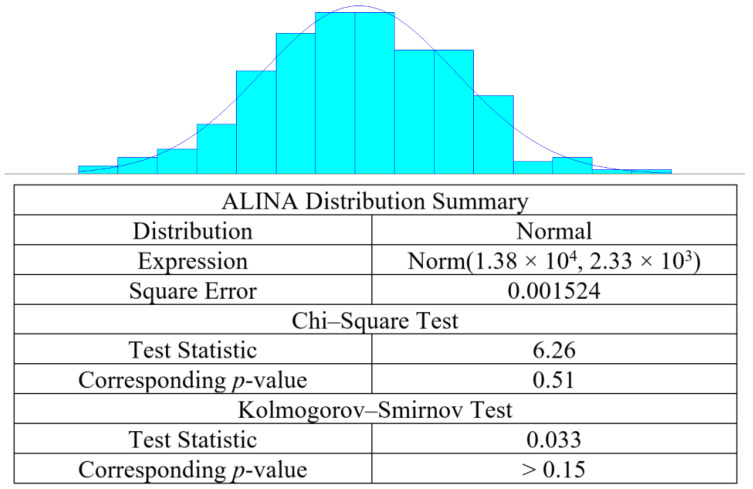
The probability distribution of the consumption of drug ALINA.

According to hospital practices, the demand for each drug is calculated through the drug lists issued by doctors after each patient sees the doctor. The main research scope of this system was the outpatient drug inventory data. All medication orders issued by the outpatient department of the case hospital on the same day were collected and consolidated as the drug demand generated in one day. The daily demand data of each drug, as collected by this study, were processed with data fitting (i.e., Arena Input Analyzer toolbox) to determine the probability distribution of daily drug demand. This study used the Arena Input Analyzer toolbox to determine the probability distribution of drug consumption based on the outpatient drug inventory data provided by the case hospital from January to December 2019. Taking ALINA as an example, according to Figure 7, the probability distribution of ALINA consumption followed a normal distribution with the mean equal to 1.38 × 10^4^ and the standard deviation equal to 2.33 × 10^3^. As the corresponding *p*-values of the chi-square test and Kolmogorov–Smirnov test were both greater than 0.05, this was a suitable distribution of the ALINA consumption probability.

**Figure 8:**
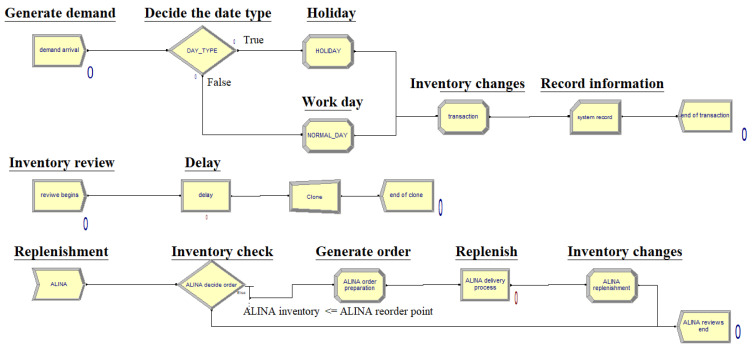
Daily check and replenishment processes of the outpatient drug inventory simulation model by using Arena.

Figure 8 shows a flowchart of the outpatient drug inventory simulation model using Arena. This study simulated the daily outpatient drug inventory checks as performed by outpatient pharmacy staff. If the drug inventory was below the minimum drug inventory after the inventory check, the replenishment process was initiated to the main pharmacy. This study simulated outpatient pharmacy staff entering the drug inventory information system to take inventory to determine whether the current drug inventory was lower than the minimum drug inventory. If yes, then the replenishment operation was carried out; otherwise, it was not. However, since the minimum packaging quantity was used by the main pharmacy of the case hospital as the unit to conduct replenishment, the method of calculating the replenishment quantity was the quotient of (maximum drug inventory - current drug inventory)/minimum packaging volume. This quotient was unconditionally rounded up and multiplied by the minimum packing quantity to obtain the drug replenishment quantity. After completing the replenishment simulation, the model in this study calculated the current drug inventory, the number of drug replenishments, and the drug replenishment quantity.

Considering that the model in this study needed to be simulated in a steady-state situation, by referring to the actual situation of multiple replenishments and the consumption of the inventory within a month, this study set the warm-up time as 30 days to ensure that the model was not affected by the initial set values. Data collection began when a steady-state situation was reached after 30 days of simulation.

According to the method in [29], the half-width of the drug consumption data, as obtained by the simulation, was divided by the average, and the initial simulation was carried out five times to obtain the result of all the quotients less than 0.05. In order to obtain a more stable result in this study, the target quotient was defined as less than 0.02. According to the method in [29], if the quotient is to be reduced by 50%, then the number of simulations must be increased by more than four times. Therefore, in this study, the number of simulations was set to 30, the simulation was performed again, and the obtained quotient was indeed less than 0.02, which met the expected value. Therefore, in order to ensure the model’s stable performance, the number of simulations in this study was set to 30.

### 4.5. System Simulation Model Verification and Validation

The simulation program logic of the system simulation model had to be verified, and the consistency of the simulation model with the actual situation had to be validated. The following made up the verification and validation of the outpatient drug inventory simulation model of this study:

(1) Verification that the logic of the simulation program logic is correct

Based on the changes in drug consumption and drug inventory, as generated by the simulation model, this study verified whether the drug consumption was calculated correctly if the drug inventory was sufficient; whether the model correctly counted the quantity of drug shortage, the number of shortages, and the quantity of drugs in stock when the drug inventory was not enough; whether the outpatient pharmacy staff in the simulated inventory check replenished the drugs according to the drug inventory policy conditions; whether the quantity of the replenishment was consistent with the logic; and whether the inventory was calculated correctly after the drug is replenished. The verification process was not carried out until all the model logics were correct.

(2) Validation on whether the model is consistent with the actual case hospital

After verifying that the logic of the simulation model was correct, this study imported the case hospital data into the model simulation and compared the simulation results with the case outpatient inventory situation. A follow-up simulation optimization study was conducted after validation. The data fitting of the outpatient drug inventory simulation model of this study was made according to the drug consumption of the case hospital. Then, the inventory obtained by the simulation was compared with the actual inventory to determine whether they were consistent with each other in order to prove that the model established in this study could represent the outpatient drug inventory of the case hospital.

This study validated the average daily drug inventory by comparing the average daily drug inventory of the actual data with the 95% confidence interval of the average inventory as obtained from 30 simulations. These results were similar to the actual average drug inventories in the outpatient pharmacy of the case hospital, which indicated that the constructed simulation model was successfully validated. Therefore, this model was sufficient to represent the current drug inventory situation in the outpatient pharmacy of the case hospital and could be used for subsequent analysis in this study.

### 4.6. Simulation Optimization for Solving Three Scenarios

This study used three scenarios for simulation analysis, namely, the current drug inventory simulation, as based on the actual set values; the individual-drug inventory optimization simulation, which optimized the simulation of individual drugs as the target; and the group-drug inventory optimization simulation, which targeted the drug groups obtained by the two-stage clustering method. According to the results obtained for the number of simulations, the optimization simulation parameter was set to 30 times.

#### 4.6.1. Scenario 1: Current Drug Inventory Simulation

This study first established a current drug inventory model with Arena for the simulation with the actual set inventory values, where the minimum inventory and inventory multipliers were adjusted by the outpatient pharmacy manager according to experience. Then, this study obtained the simulation results of each drug with a simulation length of a year, including the minimum inventory (*s*), inventory multipliers, average daily inventory cost, number of replenishments, and total drug shortages. Table 2 shows the data for Group 1.

Table 2 shows that each drug in Group 1 had its own minimum inventory (*s*) and inventory multipliers, and some drugs may have been out of stock because the set values were the parameters set based on experience. Once a drug shortage occurred, there would be corresponding measures to deal with it; thus, additional manpower and material resources would still be required. From the managers’ perspective, they would like to avoid shortages in drug inventory. Therefore, this study was based on the constraint of no drug shortages to optimize and obtain the inventory setting values for Scenarios 2 and 3.

#### 4.6.2. Scenario 2: Individual-Drug Inventory Optimization Simulation

**Figure 9:**
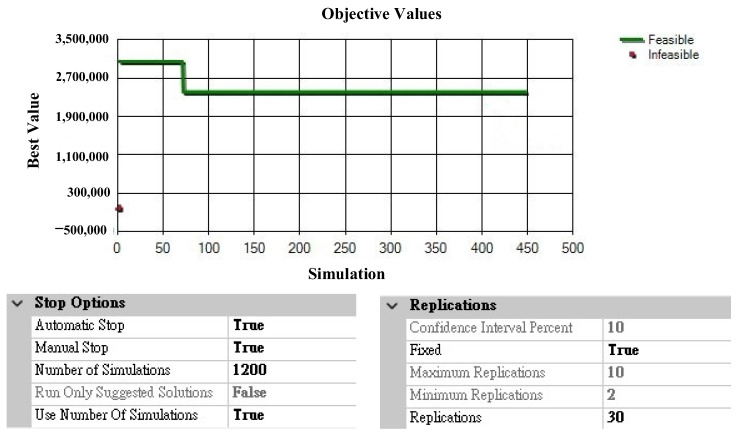
Settings for the numbers of iterations and replications.

This study used Arena OptQuest to solve the two drug inventory optimization simulation problems. The objectives and constraints were set according to each scenario, and the iteration termination condition was set to 1200. Taking Group 2 as an example, as the model failed to obtain better solutions after 50 iterations, it was automatically terminated at 450 iterations. The settings of the number of simulations and iteration times are shown in Figure 9.

(1) Objectives

**Figure 10:**
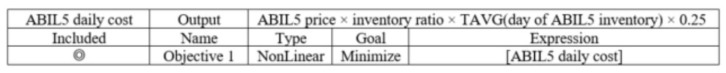
Setting values of the minimal objective function for drug ABIL5.

Taking the drug ABIL5 as an example, the setting values in Arena OptQuest are shown in Figure 10.

(2) Decision variables

**Figure 11:**
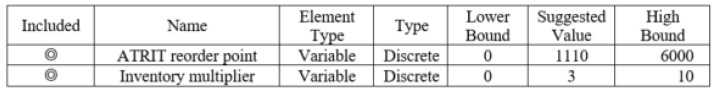
Setting values of decision variables for drug ABIL5.

Taking the drug ABIL5 as an example, the decision variables were the minimum inventory setting value of the drug and the drug inventory multiplier. The upper limit of the inventory setting value solution was set to 10 times the average consumption of the drug, the inventory multiplier was set to 10, and the variable type was discrete. The setting values in Arena OptQuest are shown in Figure 11.

(3) Constraints

**Figure 12:**
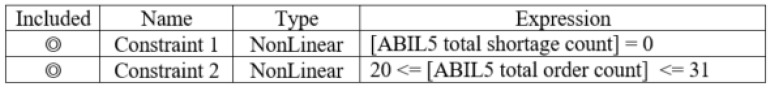
Setting values of constraints for drug ABIL5 in Scenario 2.

Taking the drug ABIL5 as an example, the constraints were divided into two parts. In order to avoid too-frequent replenishments and to ensure that the number of replenishments fell within the hospital pharmacy’s acceptable range, the constraint on the number of replenishments was set to (1 ± 20%) of the number of the current drug inventory simulation (Scenario 1). In order to ensure that the obtained optimization results would not lead to a drug shortage, the constraint of the number of drug shortages was set to be equal to 0. The setting values in Arena OptQuest are shown in Figure 12.

(4) Optimization simulation results

Taking Group 1 as an example, there was no drug shortage in the optimization results. Comparing the average daily inventory cost with that in the current drug inventory simulation showed that it dropped from NTD 631,970 to NTD 523,766, which was a decrease of 17.12%. It can be seen from the results obtained by the optimization simulation, shown in Table 3, that each drug had different values for the minimum inventory (*s*) and inventory multiplier.

#### 4.6.3. Scenario 3: Group-Drug Inventory Optimization Simulation

(1) Objectives

**Figure 13:**
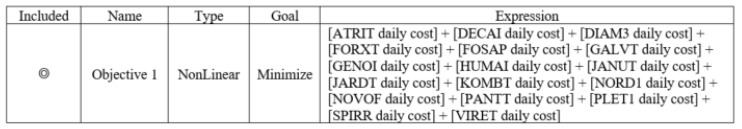
Setting values of the minimal objective function for the 17 drugs in Group 1.

The average daily inventory cost of multiple drugs in the same group was calculated with the group as a unit, and the formula was the same as that for the individual-drug inventory optimization simulation. The difference was that for group-drug inventory optimization simulation, the average daily inventory cost of multiple drugs was added as the objective. Taking Group 1 as an example, the setting values are shown in Figure 13.

(2) Decision variables

**Figure 14:**
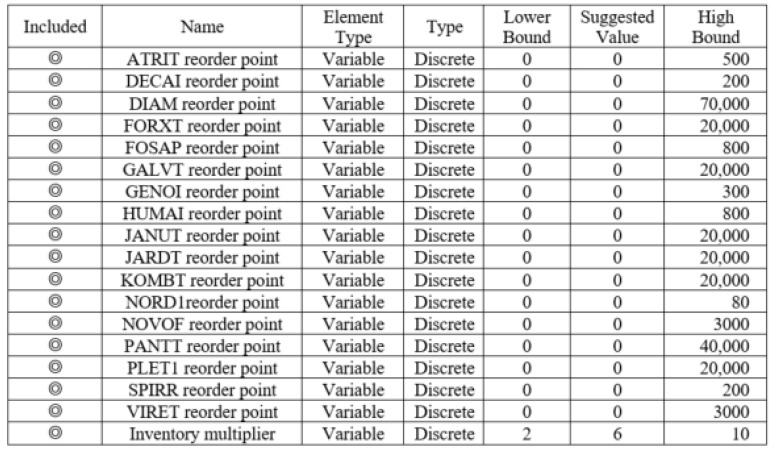
Setting values of decision variables for Group 1.

Unlike in the individual-drug inventory optimization simulation, the inventory setting value of all drugs in the same group was used as a decision variable in the group-drug inventory optimization simulation. The biggest difference was that drugs in the same group shared a drug inventory multiplier as a decision variable. As in the individual-drug inventory optimization simulation, the upper limit of the inventory setting value was set to 10 times the average consumption of the drug, the inventory multiplier was set to 10, and the variable types were all discrete. The setting values in Arena OptQuest are shown in Figure 14.

(3) Constraints

**Figure 15:**
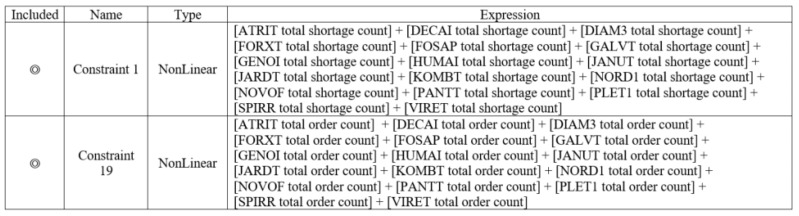
Setting values of constraints for Group 1 in Scenario 3.

Unlike the individual-drug inventory optimization simulation, if the group-drug inventory optimization simulation used the number of replenishments for individual drugs as a constraint, that would make it difficult for the model to meet the constraints for all drugs; the larger the group, the more difficult it would be to solve the problem. Therefore, the group-drug inventory optimization simulation limited the number of drug replenishments in the group unit; the total number of replenishments obtained by the current drug inventory simulation (Scenario 1) was multiplied by 1.2 times to calculate the constraint on the number of replenishments. The setting values are shown in Figure 15.

(4) Optimization simulation results

Taking Group 1 as an example, the average daily inventory cost was NTD 843,459. Compared with the current drug inventory simulation, the average daily inventory cost rose from 631,970 to NTD 843,459, which was an increase of 33.47%. The inventory multiplier of each drug obtained by the simulation optimization of Group 1 in Table 4 was the same (i.e., 2.0). The total drug shortages were the same as the individual-drug inventory optimization simulation, and both were 0.

#### 4.6.4. Result Comparisons

The results of the three scenarios studied herein are introduced for comparison as follows:

(1) Scenario 1: Current drug inventory simulation

Simulation was carried out with the inventory setting value and inventory multiplier set in the actual situation. The simulation result was close to the actual situation.

(2) Scenario 2: Individual-drug inventory optimization simulation

With the constraint of (1 ± 20%) of the number of drug replenishment times, as obtained from Scenario 1, and the zero-drug shortage setting, the minimum inventory setting value and inventory multiplier were obtained with the goal of minimizing inventory cost.

(3) Scenario 3: Group-drug inventory optimization simulation

With the group as a unit, and setting the inventory multiplier shared by a group and the drug shortage setting to 0, the minimum inventory setting value and inventory multiplier of each drug in the group was obtained by minimizing the inventory cost as the goal.

The simulation model established in this study represented the actual inventory status of the hospital outpatient pharmacy. This model was used in a group mode to explore and evaluate the simulation results of three scenarios of actual inventory in the outpatient department, the best inventory of each drug in the outpatient department, and the best inventory of the group.

**Figure 16:**
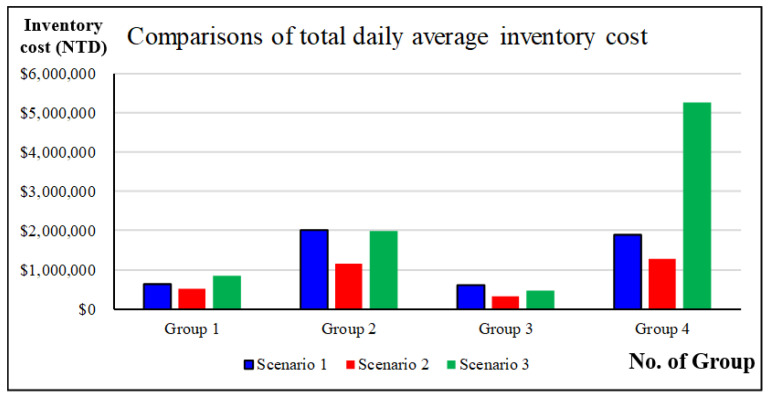
Comparison of simulation results of four drug groups for three scenarios.

Group 1: The drugs in this group belonged to the recession type and had stable, low consumption and a high chronic disease prescription ratio. There were 17 drugs in total. As shown in Figure 16, the total average daily inventory cost of outpatient drugs in the case hospital was NTD 631,970 per day in the case hospital, and the total average daily inventory cost in the individual-drug inventory optimization simulation was NTD 523,766, representing a decrease of 17.12% compared with the current situation. The total average daily inventory cost of the group-drug inventory optimization simulation was shown to be NTD 843,459, representing an increase of 33.47% compared with the current situation. In the case of no drug shortage, the average daily inventory cost for outpatient drugs in Group 1 of the individual-drug inventory optimization simulation was the minimum.

Group 2: The drugs in this group belonged to the growth type and had stable, low consumption and a high chronic disease prescription ratio. There were 60 drugs in total. As shown in Figure 16, the total average daily inventory cost of outpatient drugs in the case hospital was NTD 2,004,838, and the total average daily inventory cost in the individual-drug inventory optimization simulation was NTD 1,163,383, representing a decrease of 41.97% compared with the current situation. The total average daily inventory cost of the group-drug inventory optimization simulation was NTD 1,985,158, representing a decrease of 0.98% compared with the current situation. In the case of no drug shortage, the average daily inventory cost for outpatient drugs in Group 2 in the individual-drug inventory optimization simulation was the minimum.

Group 3: The drugs in this group belonged to the growth type and had stable, high consumption and a high chronic disease prescription ratio. There were 12 drugs in total. As shown in Figure 16, the total daily average inventory cost of outpatient drugs in the case hospital was NTD 598,939, and the total daily average inventory cost in the individual-drug inventory optimization simulation is NTD 319,536, representing a decrease of 46.65% compared with the current situation. The total average daily inventory cost in the group-drug inventory optimization simulation is NTD 472,506, representing a decrease of 21.11% compared with the current situation. In the case of no drug shortage, the average daily inventory costs for the outpatient drugs in Group 3 in Scenarios 2 and 3 were better than that in Scenario 1.

Group 4: The drugs in this group belonged to the growth type and had divergent, low consumption and a high nonchronic disease prescription ratio. There were 36 drugs in total. As shown in Figure 16, the total daily average inventory cost of outpatient drugs in the case hospital was NTD 1,898,040, and the total average daily inventory cost in the individual-drug inventory optimization simulation is NTD 1,266,578, representing a decrease of 33.27% compared to the current situation. The total average daily inventory cost of the group-drug inventory optimization simulation was NTD 5,270,024, representing an increase of 177.66% compared with the current situation. In the absence of a drug shortage, the average daily inventory cost for outpatient drugs in Group 4 of the individual-drug inventory optimization simulation was the minimum.

### 4.7. Managerial Implications

On the whole, the results obtained by the individual-drug inventory optimization simulation were the best. When the group-drug inventory optimization simulation was performed in this study, the average daily inventory cost was expected to fall between the current drug simulation and the individual-drug inventory optimization simulation; however, the results were not as expected. Only Group 2 and Group 3 were in line with the expected results, which means that, although Group 1 and Group 4 were classified into the same group, the consumption patterns of the drugs were quite different. It is therefore not suitable for management to use the same inventory policy for all groups. This also means that group-drug management of drug settings was suitable for Group 2 and Group 3, while individual-drug management was suitable for Group 1 and Group 4.

However, from a practical point of view, a simple management method to achieve maximum benefits is what managers expect. Group 2 and Group 3 could be managed via a group-drug optimization method, and outpatient pharmacy managers could quickly obtain inventory values. Moreover, the inventory cost would be better than the current situation of the original outpatient drug inventory of the case hospital, which could improve management efficiency and inventory costs. While the inventories obtained for Group 1 and Group 4 with group-drug optimization methods failed to be better than the current situation in terms of cost, inventory management could still be optimized individually. Although this would be less efficient than group-drug management, it could still achieve better inventory costs than the current situation.

## 5. Conclusions and Future Research

### 5.1. Conclusions

This study used 125 drugs to conduct the two-stage clustering method. The outpatient drug simulation optimization and individual- and group-drug inventory optimization simulations were used to solve the proposed problem. The results shown in Figure 16 revealed that the outpatient drug optimization methods (Scenario 2) were suitable for each drug, and that the group-drug inventory optimization simulation (Scenario 3) was limited to the same inventory multipliers used by each drug in the same group to find the minimum inventory. Only Group 2 and Group 3 had better average daily inventory cost of outpatient drugs in Scenario 3 than in the case hospital (Scenario 1). However, although the optimization method for each outpatient drug was the relatively better method, it would still take a great deal of time to find a near-optimal solution for each drug. As the number of drugs increases, the construction of the simulation model and the solution time requires a heavier burden.

This study explored the management of drugs in a group-drug manner to determine a fixed rule that could be applied to a group of drugs to improve the efficiency of management and reflect good inventory costs. The group-drug inventory optimization simulation was faster than the individual-drug inventory optimization simulation for outpatient clinics, as it had the constraint that the inventory multipliers must be equal and all drugs in the same group must be solved simultaneously. Although the group-drug inventory optimization simulation obtained better results in only two groups (Group 2 and Group 3), it was still helpful. In the future, when drugs belong to these two groups, the group-drug inventory optimization simulation could be directly performed to obtain a better inventory, while the other two groups could be managed by the individual-drug inventory optimization simulation of each drug in the outpatient department.

The contributions of this study are described as follows. This study proposes a novel simulation model to analyze a large number of different types of drugs for inventory setting optimization. Using the *K*-means clustering algorithm in the model was helpful and effective to reduce the time for obtaining the optimal inventory solutions. In practice, our model could help hospitals to control drug inventories and costs and enhance drug management efficiency.

### 5.2. Limitations of This Research and Future Studies

The research scope of this study focused on the case outpatient pharmacy as the study subject. In practice, each hospital owns several pharmacies, such as outpatient, inpatient, emergency, and chemotherapy. Cross-pharmacy drug cooperation prevails for pharmacy managers in order to avoid high inventory costs and drug shortages. Therefore, extending a single pharmacy’s results to multiple pharmacies’ applications is a critical challenge for researchers. The problem of designing a good mechanism for cross-pharmacy drug cooperation merits further research.

In the process of this study, three suggestions were put forward as reference directions for future research based on the research hypotheses and with consideration of the constraints encountered:

(1) Consider adding the drug inventory model of the main pharmacy and other subpharmacies

Regarding the pharmacy department of the case hospital, the outpatient drug inventory simulation model, as constructed by this study, was the drug inventory model for only a single subpharmacy and did not consider the interactive operations of drug allocations with other subpharmacies (i.e., inpatient, emergency, and chemotherapy). Therefore, if future research can extend the constructed model to other subpharmacy models, the extended model will be more practical and referential.

(2) Consider different drug inventory policies

Different drug inventory policies may result in different drug inventory operations and quantities. In addition to finding the best maximum and minimum inventory levels for each drug in the drug inventory policy (*s*, *S*) as based on the current situation of the case, future research can explore other drug inventory policies, such as the quantitative ordering system (*s*, *Q*), regular ordering system (*T*, *S*), and comprehensive ordering system (*s*, *T*, *S*), to discuss the differences in the costs of each drug inventory policy and determine the drug inventory policy that is most suitable for the case hospital.

(3) Improve clustering accuracy

This study divided the data into four groups using a two-stage clustering method and performed a group-drug inventory optimization simulation. It was concluded that this method was applicable to two groups but not to the other two groups. This study found that the consumption patterns of the drugs were not very similar in the two groups in which the group-drug inventory optimization simulation was applicable, which led to this result. Future studies can consider other different drug-related data groups to obtain drug groups with more similarities.

## Figures and Tables

**Table 1:** Basic information of drug inventory setting.

TRACT	
Inventory day	5
Inventory multiplier	3
Consumption in the previous month	51,784
Average daily consumption	2534
Minimum inventory	12,670
Maximum inventory	38,010
Minimum manual inventory	4900
Maximum manual inventory	33,460

**Table 2:** Results for 17 drugs in Group 1 for Scenario 1.

Group 1		Simulation Results				
Drug No.	Drug Code	Minimum Inventory (*s*)	Inventory Multiplier	Average Daily Inventory Cost	Number of Replenishments	Total Drug Shortages
1	ATRIT	90	3.0	18,617	53	54
2	DECAI	28	1.5	28,940	81	0
3	DIAM3	12,934	3.0	12,206	32	0
4	FORXT	2030	2.2	21,411	61	0
5	FOSAP	157	3.0	12,912	31	0
6	GALVT	2285	3.0	11,909	36	0
7	GENOI	55	2.2	43,073	53	30
8	HUMAI	147	3.0	222,842	8	0
9	JANUT	2745	2.2	25,162	56	0
10	JARDT	2770	2.2	30,697	64	0
11	KOMBT	3725	2.2	25,886	53	0
12	NORD1	15	2.2	32,698	56	1
13	NOVOF	445	2.2	39,505	51	0
14	PANTT	7725	2.2	35,340	61	0
15	PLET1	2395	3.0	14,636	30	0
16	SPIRR	40	2.2	23,680	53	0
17	VIRET	595	2.2	32,456	100	2231
Total				631,970	879	2316

**Table 3:** Results for 17 drugs in Group 1 for Scenario 2.

Group 1	Simulation Results				
Drug Code	Minimum Inventory (*s*)	Inventory Multiplier	Average Daily Inventory Cost	Number of Replenishment	Total Drug Shortage
ATRIT	104	2.6	18,998	57	0
DECAI	21	1.5	21,311	93	0
DIAM3	4200	5.8	6514	39	0
FORXT	1200	2.6	13,500	73	0
FOSAP	77	4.1	7797	38	0
GALVT	700	8.3	7998	32	0
GENOI	72	1.9	51,402	54	0
HUMAI	94	3.0	217,569	10	0
JANUT	1200	3.3	13,943	64	0
JARDT	1500	2.7	18,201	77	0
KOMBT	1300	3.9	13,389	61	0
NORD1	14	2.0	28,018	67	0
NOVOF	190	3.3	21,772	60	0
PANTT	3300	3.2	18,285	73	0
PLET1	1000	4.7	8465	36	0
SPIRR	19	3.2	13,851	59	0
VIRET	920	1.6	42,753	117	0
Total			523,766	1010	0

**Table 4:** Results for 17 drugs in Group 1 for Scenario 3.

Group 1	Simulation Results				
Drug Code	Minimum Inventory (*s*)	Inventory Multiplier	Average Daily Inventory Cost	Number of Replenishments	Total Drug Shortages
ATRIT	400	2.0	62,506	26	0
DECAI	28	2.0	35,240	49	0
DIAM3	27,000	2.0	19,521	31	0
FORXT	9000	2.0	95,721	19	0
FOSAP	140	2.0	8371	64	0
GALVT	4000	2.0	15,952	41	0
GENOI	160	2.0	122,335	26	0
HUMAI	280	2.0	24,180	38	0
JANUT	12,000	2.0	110,107	17	0
JARDT	3000	2.0	31,147	70	0
KOMBT	4000	2.0	26,052	59	0
NORD1	19	2.0	39,187	52	0
NOVOF	220	2.0	16,400	108	0
PANTT	18,000	2.0	81,235	34	0
PLET1	10,000	2.0	47,630	15	0
SPIRR	30	2.0	15,951	79	0
VIRET	1600	2.0	91,925	53	0
Total			843,459	828	0

## Data Availability

Not applicable.

## References

[1] Buschiazzo M., Mula J., Campuzano-Bolarin F. (2020). Simulation optimization for the inventory management of healthcare supplies. Int. J. Simul. Model..

[2] Fragapane G.I., Zhang C., Sgarbossa F., Strandhagen J.O. (2019). An agent-based simulation approach to model hospital logistics. Int. J. Simul. Model..

[3] Abu Zwaida T., Pham C., Beauregard Y. (2021). Optimization of inventory management to prevent drug shortages in the hospital supply chain. Appl. Sci..

[4] Du M., Luo J.W., Wang S.P., Liu S. (2020). Genetic algorithm combined with BP neural network in hospital drug inventory management system. Neural Comput. Appl..

[5] Fernandez M.I., Chanfreut P., Jurado I., Maestre J.M. (2021). A data-based model predictive decision support system for inventory management in hospitals. IEEE J. Biomed. Health Inform..

[6] Gebicki M., Mooney E., Chen S.J., Mazur L.M. (2014). Evaluation of hospital medication inventory policies. Health Care Manag. Sci..

[7] Antonoglou D., Kastanioti C., Niakas D. (2017). ABC and VED analysis of medical materials of a general military hospital in Greece. J. Health Manag..

[8] Nigah R., Devnani M., Gupta A.K. (2010). ABC and VED analysis of the pharmacy store of a tertiary care teaching, research and referral healthcare institute of India. J. Young Pharm..

[9] Saha E., Ray P.K. (2019). Modelling and analysis of inventory management systems in healthcare: A review and reflections. Comput. Ind. Eng..

[10] Saedi S., Kundakcioglu O.E., Henry A.C. (2016). Mitigating the impact of drug shortages for a healthcare facility: An inventory management approach. Eur. J. Oper. Res..

[11] Azghandi R., Griffin J., Jalali M.S. (2018). Minimization of drug shortages in pharmaceutical supply chains: A simulation-based analysis of drug recall patterns and inventory policies. Complexity.

[12] Maestre J.M., Fernandez M.I., Jurado T. (2018). An application of economic model predictive control to inventory management in hospitals. Control Eng. Pract..

[13] Al-Fandi L.M.D., Obaid A.A.B., Alfailakawi B.I., Alsubaiei H.A., Khudhair S.A. (2019). A simulation study to determine the parameters of medicine inventory policy. Proc. Est. Acad. Sci..

[14] Fogarty D.W., Hoffmann T.R. (1983). Production and Inventory Management.

[15] Kelle P., Woosley J., Schneider H. (2012). Pharmaceutical supply chain specifics and inventory solutions for a hospital case. Oper. Res. Health Care.

[16] Dong F.G., Liu H.M., Lu B.D. (2012). Agent-based simulation model of single point inventory system. Syst. Eng. Procedia.

[17] Guerrero W.J., Yeung T.G., Gueret C. (2013). Joint-optimization of inventory policies on a multi-product multi-echelon pharmaceutical system with batching and ordering constraints. Eur. J. Oper. Res..

[18] Nematollahi M., Hosseini-Motlagh S.M., Ignatius J., Goh M., Nia M.S. (2018). Coordinating a socially responsible pharmaceutical supply chain under periodic review replenishment policies. J. Clean. Prod..

[19] Chen J.X., Liang L., Yao D.Q. (2020). An analysis of inventory policies for substitute pharmaceuticals with purchasing or selling constraints. Int. Trans. Oper. Res..

[20] Galli L., Levato T., Schoen F., Tigli L. (2021). Prescriptive analytics for inventory management in health. J. Oper. Res. Soc..

[21] Lee M.L., Park I., Park D.U., Park C. (2017). Constrained ranking and selection for operations of an emergency department. Int. J. Simul. Model..

[22] Chen P.S., Chen G.Y.H., Liu L.W., Zheng C.P., Huang W.T. (2022). Using simulation optimization to solve patient appointment scheduling and examination room assignment problems for patients undergoing ultrasound examination. Healthcare.

[23] Asgary A., Najafabadi M.M., Karsseboom R., Wu J.H. (2020). A drive-through simulation tool for mass vaccination during COVID-19 pandemic. Healthcare.

[24] Lee S., Lee Y.H. (2020). Improving emergency department efficiency by patient scheduling using deep reinforcement learning. Healthcare.

[25] Wu I.C., Lin Y.C., Yien H.W., Shih F.Y. (2020). Constructing constraint-based simulation system for creating emergency evacuation plans: A case of an outpatient chemotherapy area at a cancer medical center. Healthcare.

[26] Liu Z.Y. (2017). The anti-hepatitis drug use effect and inventory management optimization from the perspective of hospital drug supply chain. Pak. J. Pharm. Sci..

[27] Alves R.J.V., Etges A., Neto G.B., Polanczyk C.A. (2018). Activity-based costing and time-driven activity-based costing for assessing the costs of cancer prevention, diagnosis, and treatment: A systematic review of the literature. Value Health Reg. Issues.

[28] Abbas S.A., Aslam A., Rehman A.U., Abbasi W.A., Arif S., Kazmi S.Z.H. (2020). K-means and k-medoids: Cluster analysis on birth data collected in city Muzaffarabad, Kashmir. IEEE Access.

[29] Kelton W.D., Sadowski R., Zupick N. (2015). Simulation with Arena.

